# Supplementary material for: Phylogenomic and Evolutionary Analyses Reveal Diversifications of SET-Domain Proteins in Fungi
Source: J Fungi (Basel). 2022 Nov 2;8(11):1159. doi: 10.3390/jof8111159 (PMC9692433; doi:10.3390/jof8111159)
Supplement: Supplementary file 1 [file jof-08-01159-s001.zip › jof-1956913-supplementary.pdf]

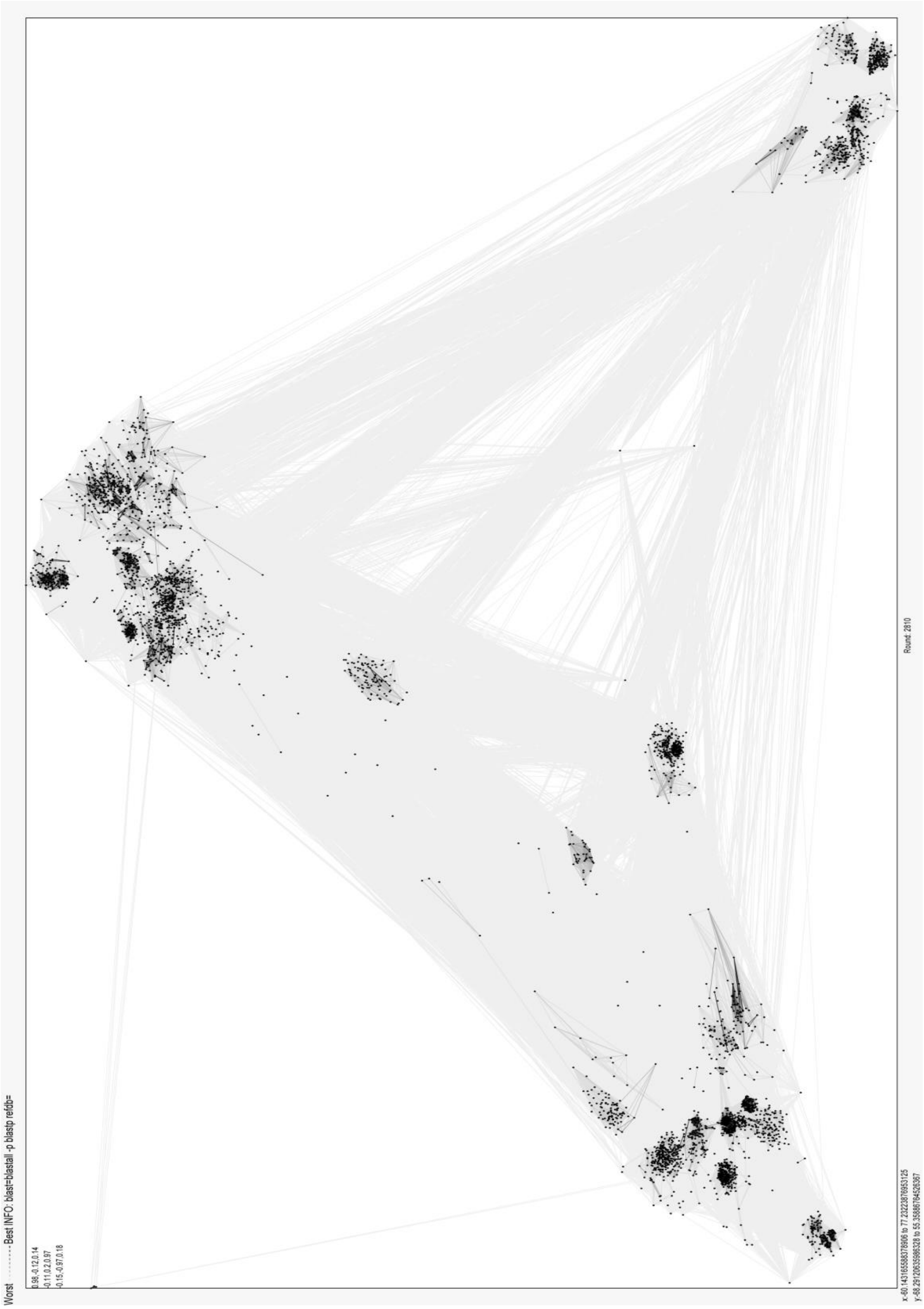

Figure S1. CLANS clustering of 4171 SET domain sequences obtained from 229 whole genome sequenced fungal species. A Java-based software program, CLANS, was used to elucidate the relationships between and within subfamilies of the putative 4172 SET domain sequences from 229 fungal genomes.

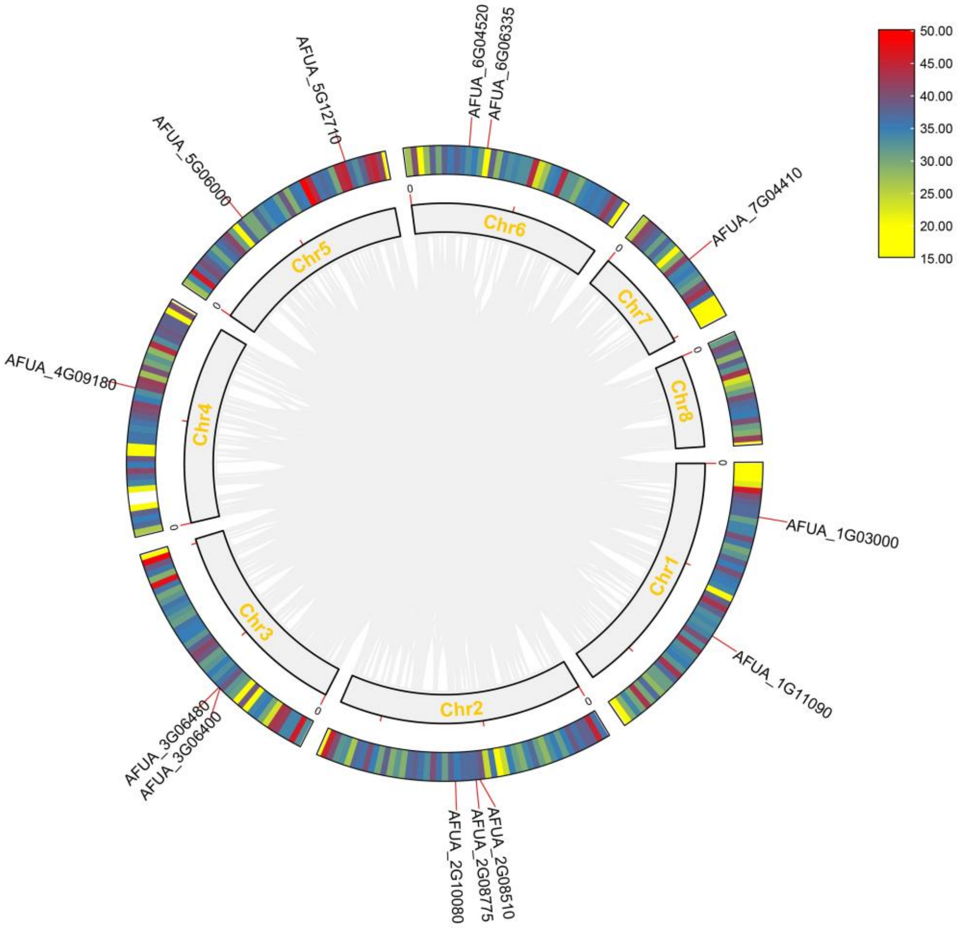

Figure S2. Chromosomal distribution of the SET-domain containing genes in A. fumigatus genome. 13 SET-domain genes are unevenly distributed on the 8 chromosomes of A. fumigatus genome, and the number of genes on each chromosome is irrelevant to chromosome size.

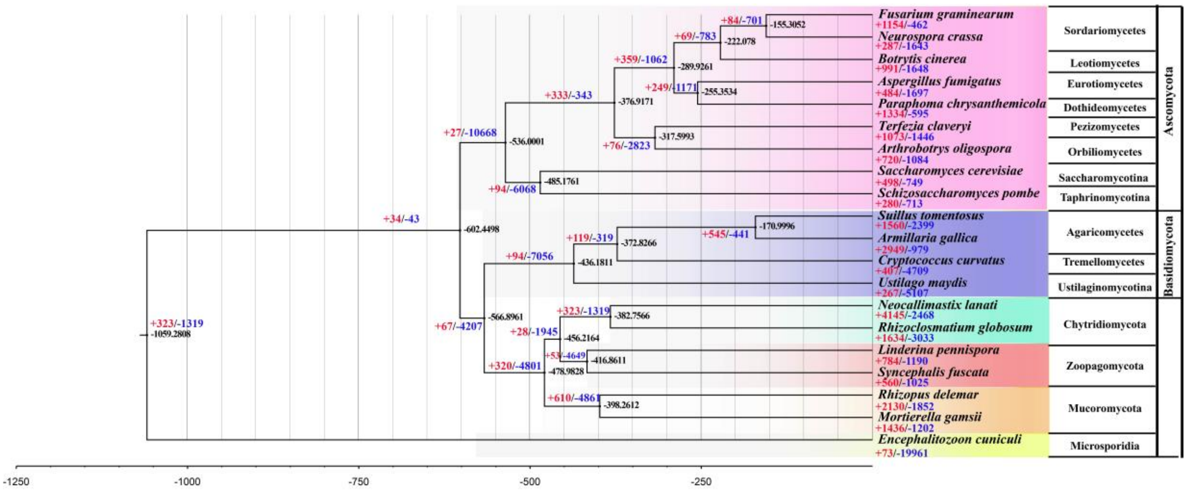

Figure S3. Various expansions and contractions among the fungal lineages using CAFE analysis. The numbers present on each node correspond to the expansion and contraction numbers of orthologues genes predicted by CAFE.
